# Anti-virulence potential of eugenol-rich fraction of *Syzygium aromaticum *against multidrug resistant uropathogens isolated from catheterized patients

**Published:** 2018

**Authors:** Prasanth Rathinam, Pragasam Viswanathan

**Affiliations:** *Renal Research Lab, Centre for Bio Medical Research, School of Bio Sciences and Technology, Vellore Institute of Technology, Vellore, India*

**Keywords:** Syzygium aromaticum (L.), Anti-virulence agent, Quorum sensing, Eugenol, Pseudomonas aeruginosa, CAUTIs

## Abstract

**Objective:**

Considering the emergence of biofilm-associated pathogens with multidrug resistance, the objective(s) of the present study was to evaluate the anti-virulence property of *Syzygium aromaticum *extracts/fractions against 2 multidrug-resistant catheter isolates.

**Materials and Methods:**

Pulverized clove buds were subjected to bioactivity-guided isolation to identify the bioactive extract/fraction(s) with significant anti-virulence property. The clove bud powder was subjected to Soxhlet extraction and all these extracts were investigated in terms of anti-virulent efficacy using initial readout assays. Bioassay-guided partial purification was performed through column and size exclusion chromatography. Detailed testing of the anti-virulence and anti-biofilm activity of sub-minimum inhibitory concentration (sub-MIC) levels of the active fraction, was performed besides the cytotoxicity profiling in RAW 264.7 cells.

**Results:**

Gas chromatography-mass spectrometry (GC-MS) analysis of the clove bioactive fraction-2 (CBF-2), done after the bioassay-guided fractionation, revealed eugenol as the major bioactive ingredient present in CBF-2. Reduced mRNA levels of virulence factor genes after CBF-2 (700 μg/ml) treatment correlated well with the respective phenotypic assays (p<0.001). Similarly, CBF-2 (700 μg/ml) treatment exhibited significantly low mRNA levels of quorum sensing (QS) receptor genes compared to their cognate synthase genes (p<0.001). Crystal violet staining and scanning electron micrographs of CBF-2-treated biofilms showed lesser macrocolonies with remarkably simplified architecture. Cytotoxic evaluation of CBF-2 suggested a minute reduction in viability even at the highest tested concentration (1600 μg/ml, p<0.05).

**Conclusion:**

The present study indicated that eugenol-rich CBF-2 has potent anti-virulence and anti-biofilm activity against urinary catheter isolates and can be regarded as an alternative for treatment of catheter-associated urinary tract infections.

## Introduction

*Pseudomonas aeruginosa,* as an opportunistic pathogen, is considered one of the major causes of nosocomial infections like catheter-associated urinary tract infections (CAUTIs) (Mittal et al., 2009 ▶). Quorum sensing (QS) inhibitors as anti-virulent drugs were of most interest since they do not pose selective pressure and may reduce rapid emergence of drug-resistant pathogens (Sarabhai et al., 2013 ▶). Additionally, dietary compounds like curcumin from turmeric, ajoene from garlic and iberin from horseradish were found to have anti-virulence property and considered safe since they cause less/no toxicity to mammalian tissues upon consumption (Sarabhai et al., 2013 ▶). Predominantly, anti-QS property of phyto-compounds has been tested with the help of engineered reporter strains and laboratory reference strains even though under stress conditions, these strains behave unusually as evidenced by variations in expression patterns (Van Deldenand Iglewski, 1998 ▶, García-Contreras et al., 2015 ▶). 

Though no resistance was expected to be developed against anti-QS compounds, recent studies in *P. aeruginosa* with mutations on efflux have identified resistance against one of the potent QS inhibitors, brominated furanone C-30 (García -Contreras et al., 2015 ▶, García-Contreras et al., 2013 ▶). Additional clinical evidence on the ability of clinical strains to evolve resistance towards QS inhibitors was proposed (Gibot, 2004 ▶). Based on the literature, *P. aeruginosa* isolates from urine, blood and urinary catheter specimens of cystic fibrosis patients were found to be resistant towards QS inhibitors like C-30 and 5-flurouracil (García -Contreras et al., 2015, García-Contreras et al., 2013). These findings highlight the importance of testing QS inhibitors not only in laboratory model strains like *P. aeruginosa* PAO1 but also in clinical isolates that potentially could have the ability to neutralize QS inhibitors’ effects.

*Syzygium aromaticum* (Clove), belonging to the Myrtaceae family has been widely recognized for its medicinal and culinary properties. The dried clove buds are rich in polyphenols and essential oils and have been used in Ayurveda, Chinese medicine and dentistry (Kong et al., 2014 ▶). Clove oil (essential oil from clove) at sub-MIC levels reported to repress QS-associated characters in reporter strains (Khan et al., 2009 ▶). Similarly, clove bud extracts exhibited anti-QS property in reporter strains and *P. aeruginosa* PAO1 (Krishnan et al., 2012 ▶). The present study evaluated the anti- virulence potential of clove bud extracts against 2 multidrug-resistant clinical isolates from patients having CAUTIs (i.e. RRLP1 and RRLP2) besides PAO1. The clove bioactive fraction (CBF-2), identified through bioassay-guided fractionation and characterized by GC-MS analysis, was analyzed in detail for its anti- virulence and anti-biofilm efficacy. *In vitro* toxicity analysis of the active fraction was also performed in mouse macrophage cell line (RAW 264.7). The findings of this study can be further applied to develop anti-pathogenic drugs to combat biofilm-assisted infections in the clinical setting.

## Materials and Methods

**Bacterial strains and culture conditions**

The bacterial strains used in this study were *P. aeruginosa* PAO1 (MTCC 3541) and 2 clinical isolates - *P. aeruginosa* RRLP1 (GenBank ID: KR149278) and *P. aeruginosa *RRLP2 (GenBank ID: KT309033), isolated from patients having CAUTIs, referring to Government Vellore Medical College and Hospital, Adukkamparai, India. The clinical isolates were identified with an intact LasI/R and RhlI/R QS systems and were able to produce quorum-regulated virulence factors (VFs) (Rathinam and Viswanathan, 2014). All the test organisms were cultured in Luria-Bertani broth (Himedia Laboratories Pvt Ltd, Bangalore, India, pH 7.0) and maintained under 120 rpm at 37 °C. All the bacterial cultures were sub-cultured until an optical density (OD) of 0.4 was reached at 600 nm. 

**Preparation of plant material and extraction**

The obtained *S. aromaticum* (Clove) buds were washed, shade-dried, pulverized and stored. The voucher specimens were identified by Dr. Angeline Vijayakumari, Head of Department of Botany, Voorhees College, Vellore, Tamil Nadu, India and deposited in Voorhees College Herbarium (Voucher No. VITRRL002/2013). The powdered clove buds (100 g) were progressively extracted with 500 ml of solvents (petroleum ether, dichloromethane, ethyl acetate and methanol). These extracts were filtered and evaporated under vacuum and stored at 4°C for further studies.

**Primary assays for detecting the anti-QS activity of extracts**

**Figure 1:**
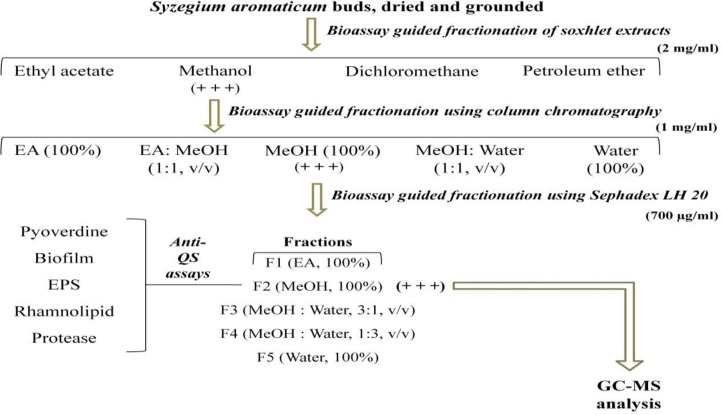
Schematic presentation of bioassay guided fractionation and characterization of clove bud extracts. Ethyl acetate, MeOH-Methanol, EPS-Extracellular polysaccharides, (+ + +)-extract/fraction with significant results in readout assays.

The extracts were subjected to bioassay-guided partial purification to identify the potent anti-QS fraction(s) as presented in Figure 1. Each plant extract (2 mg/ml) was tested against the QS-regulated virulence factors (VFs); pyoverdine and biofilm production by *P. aeruginosa* PAO1 was quantified as indicated by the previous protocol(s), by initial readout assays before further fractionation (Adonizio et al., 2008, Packiavathy et al., 2012). 

**Separation of bioactive fraction(s) from the methanol extract of** ***S. aromaticum***

**Column chromatography**

The methanol extract was subjected to silica column chromatography (200-300 mesh size with 50 cm length and i.d. 2 cm). Methanol extract (5 mg/ml), was subsequently eluted with ethyl acetate (100%), ethyl acetate: methanol (1:1 v/v), 100% methanol, methanol: water (1:1 v/v) and 100% water at a flow rate of 2 ml/min. After complete elution of the loaded sample, each fraction (15 ml) was collected, dried and subsequently assayed for anti-QS activity, at 1 mg/ml. 

**Size exclusion chromatography (SEC)**

Methanol fraction (5 mg/ml) was loaded onto Sephadex LH 20 column (20 cm length and i.d. 2 cm) and successively eluted with methanol (v/v) (100, 50, 30, 20 and 10%) and 100% water. Individual fractions (20 ml) were collected, dried, re-suspended (700 µg/ml) and tested for anti-QS properties (Packiavathy et al., 2012).

**Minimum inhibitory concentration (MIC) determination of clove active fraction (CBF-2) and growth curve analysis**

The MIC was assessed against the test organisms (PAO1, RRLP1 and RRLP2), using the micro-dilution method according to the Clinical and Laboratory Standards Institute guidelines (Wikler, 2009). All further experiments in the study were performed at sub-MIC levels of CBF-2. Dose-response growth curves of the test organisms were plotted at sub-MIC levels of CBF-2.

**GC-MS Analysis of CBF-2**

GC-MS analysis was performed to detect the major compound(s) present in CBF-2 as described in the previous study (Unnikrishnan et al., 2014). The compounds were identified based on their molecular structure and molecular mass and by comparing the calculated fragment ratio of resolved spectra with that obtained from the library. Spectral data were interpreted using the National Institute Standard and Technology (NIST) database. Samples were tested in duplicates and the percentage composition was calculated by the normalization method from the GC peak areas, assuming identical mass response factors for all compounds.

**Quantitative VFs inhibition by test organisms**

LasB elastase production by the test organisms after CBF-2 treatment, was estimated in the culture supernatants using elastin Congo red method (Adonizio et al., 2009). Activity was expressed as the change in the optical density per g protein. Rhamnolipids, synthesized by all the test organisms, with and without CBF-2 were extracted and quantified as described previously (Abraham et al., 2011). Quantification of rhamnolipids was performed spectrophotometrically at 421 nm. LasA protease activity was determined by measuring the ability of test organisms’ culture supernatants to lyse heat-killed *Staphylococcus aureus* cells. Quantification of protease was done as the change in the optical density per hour per µg of protein (Adonizio et al., 2009). All the results were represented as % reduction in protease production. The percentage inhibition was calculated using the following formula:

%inhibition= ((Control OD–Test OD) /Control OD) ×100

**Expression analysis of QS and virulence genes in test organisms**

Effects of CBF-2 (700 μg/ml) on the expression of QS and VFs genes were evaluated using quantitative PCR (RT-qPCR) (Sarabhai et al., 2013). Organisms were treated with/without CBF-2 and total cellular RNA was converted to cDNA according to the manufacturer’s protocol (Thermo Scientific, Bangalore, India). In 10 µl reaction mixture, 5 µl of SYBER green master mix (TaKaRa Bio Inc., CA, USA), 100 ng of cDNA, 5 µM of primers of the target genes - QS genes; *lasR* and *rhlR* (codes for QS receptors), *lasI* and *rhlI* (codes for QS signal synthase), VF genes; *lasA*, *rhlB*, *lasB*, *pvdA *and internal housekeeping gene (16s rRNA) (Sarabhai et al., 2013) were added. The RT qPCR was done using a two-step PCR program: 10 min at 95 °C (denaturation) and 30 cycles: 10 sec at 95 °C and 60 sec at 60 °C. Results are expressed as relative expression of gene (RQ); calculated by 2^-ΔΔct^. The percent reduction was calculated as (1-RQ) × 100, compared to the control (without CBF-2). The list of primers used is presented as Supplementary Table 1. 

**Biofilm and extracellular polysaccharides (EPS) inhibition in test organisms**

Test organisms were allowed to form biofilms over glass slides (2×2 cm) immersed in Luria-Bertani broth with or without CBF-2 and the quantification of biofilm was performed by crystal violet (CV) quantification method and EPS quantification was performed by total carbohydrate estimation method (Packiavathy et al., 2012). The obtained results were represented as % reduction and calculated based on the following formula:

%inhibition= ((Control OD–Test OD) /Control OD) ×100

The biofilm formation by the test organisms, with CBF-2 (PAO1 and RRLP1-700 µg/ml as well as RRLP2-600 µg/ml) was visualized under light microscopy (Packiavathy et al., 2012). Briefly, 1% of the overnight-grown cultures of the test organisms, was added to 2 ml of fresh media containing a glass slide (1×1cm) in a 6 well micro titer plate (MTP) with and without CBF-2 and allowed to develop biofilm. After the incubation period, the glass slides were washed with distilled water to remove the planktonic cells and subsequently stained with CV (0.2% w/v) solution and visualized using a microscope at the magnification of 40X.

**Acridine orange/Ethidium bromide (AO/EB) staining of biofilm-associated microbes**

All the test organisms were allowed to form biofilm on glass slides with and without CBF-2 (700 μg/ml), as described previously (Packiavathy et al., 2012). After the incubation period, the a suspension of biofilm-associated cells were prepared and stained using AO/EB (4 µg/ml) of 1:1 ratio and incubated for 15 min at 37 °C in the dark; next, 2 µl of it was visualized under a epifluorescent microscope (WESWOX OPTIK-FM3000 equipped with WESWOX DG-140 digital high resolution camera, India), at the magnification of 40X, to differentiate live and dead cells (Ćurčić et al., 2012).

**Biofilm eradication property of CBF-2 in urinary catheters assessed by scanning electron microscopy (SEM)**

To ensure the ability of CBF-2 in disrupting the matured biofilm produced by test pathogens, biofilm disruption assay was performed using a previously reported method (Abraham et al., 2011). Briefly, overnight-grown biofilms of the test organisms on urinary catheter pieces were treated with CBF-2 (700 μg/ml) for 5-6 hr. After the incubation period, these catheter pieces were washed, processed and observed under scanning electron microscope (ZEISS-EVO18, India). 

**Toxicity of CBF-2 (** ***in vitro*** **)**

*MTT assay*

 The cytotoxic effect of CBF-2 was evaluated in mouse macrophage cell line (RAW 264.7) using previously reported protocols (Unnikrishnan et al., 2014). The rate of cell viability was measured at 570 nm using a spectrophotometer. The data was presented as % viability ± SD (n=6) and calculated using the formula:

%Viability= (Control OD–Test OD) /Control OD) ×100

*Lactate dehydrogenase (LDH) assay*

RAW 264.7 cells were seeded at a density of 5×10^5^ cells/ml and allowed to attach for 24 hr in 300 𝜇l of medium and incubated at 37 °C with 5% CO_2_. After 24 hr, CBF-2 was added to the medium (0, 100, 200, 400, 800 and 1600 µg/ml) and incubated for 24 hr. Triton X-100 at 0.1% (w/v) was used as positive control and LDH release was considered to be 100%. Cells added to the media without any test compound, were kept as growth control. The concentration of LDH released was measured using an LDH cytotoxic test kit (Coral clinical systems, India) according to the manufacturer’s protocol. The data was presented as %leakage±SD (n=6) as calculated using the formula:

%Leakage= ((Positive Control OD–Test OD) /Positive Control OD) ×100

*Estimation of Thiobarbituric Acid-Reacting Substances (TBARS) assay*

TBARS levels were quantified spectrophotometrically as described previously (Janero, 1990). Here, 0.5% (v/v) hydrogen peroxide was used as positive control and cells grown without test compound were kept as growth control. The levels of TBARS in the cell-free supernatants, after CBF-2 treatment, were compared with the growth control and data was represented as nmol of TBARS per ml of supernatant±SD (n=6).

**Statistical analysis**

All experiments were performed independently and the data was represented as Mean±Standard Deviation (SD), and evaluated using GraphPad Prism 5.A p value<0.05 was considered significant. All the results of the virulence factors estimation assay were analyzed by two way ANOVA with Tukey’s multiple comparison posttest. Comparisons were performed concerning the virulence factor production at different CBF-2 concentrations as ‘a’-500 µg/ml vs 700 µg/ml, ‘b’-600 µg/ml vs 700 µg/ml, ‘c’- 500 µg/ml vs 600 µg/ml. Gene expression results were analyzed by two way ANOVA with Sidak’s multiple comparison posttest. Comparisons were performed as respective genes of CBF-2 untreated vs CBF-2-treated. The toxicity profiling results were analyzed by one way ANOVA with Dunnett’s multiple comparison posttest and the comparisons were performed vs control (CBF-2, 0.0 μg/ml).

## Results

**Bioassay-guided fractionation and GC-MS analysis**

**Figure 2:**
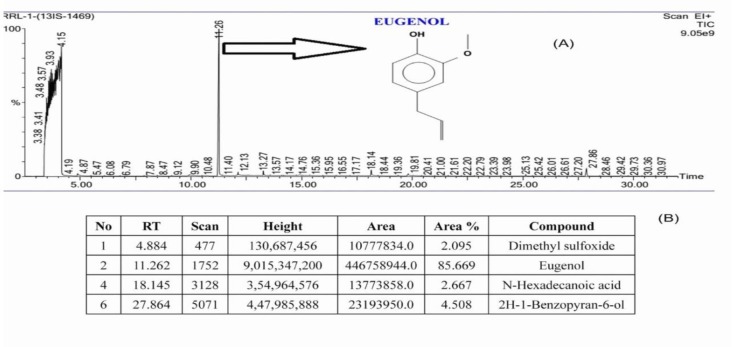
GC-MS analysis of CBF-2. (A) Gas chromatogram of CBF-2, detecting eugenol (RT=11.262) (B) The normalized % Table of major components present in CBF-2.

Bioassay guided partial purification of dried *S. aromaticum* bud extracts were performed, as per the scheme presented in Figure 1, which led to separation of clove bioactive fraction-2 (CBF-2) with significant initial readout assay results. Clove bioactive fraction-2 (CBF-2) at 700 μg/ml exhibited notable anti-QS activity indicated by significant reduction in biofilm and pyoverdine production (52 and 40%, respectively) by PAO1, p<0.001 (Table 1). Gas Chromatography-Mass Spectrometry (GC-MS) analysis of CBF-2 with subsequent matching of retention time (RT) and mass spectra comparison with NIST library revealed Eugenol, RT-11.26 (area%: 88.67), N-Hexanoic acid, RT-18.14 (area %: 2.68) and 2H-1-Benzopyran-6-ol, RT-27.86 (area%: 4.5) as major compounds, besides many other minor components, as shown in Figure 2.

**Table 1:** Bioassay guided fraction of *S. aromaticum* extract(s) on biofilm and pyoverdine production by* P. aeruginosa* PAO1.

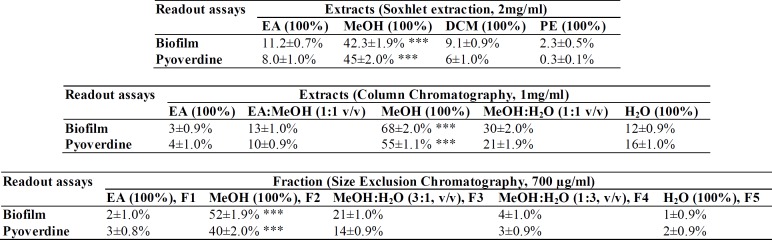

Percentage inhibition of respective virulence factors are presented as mean±SD (n=6). The results were analyzed by one way ANOVA with Dunnett’s multiple comparison posttest. Comparisons were performed with Control (Untreated *P. aeruginosa* PAO1 cells) (***p<0.001). EA: Ethyl acetate; MeOH: -Methanol; DCM: Dichloromethane, and PE: Petroleum ether.

**Determination of sub-MIC levels and growth curve study**

The antibiotic susceptibility pattern of the clinical isolates was assessed (Supplementary Table 2) and clarified as multidrug-resistant (MDR) as per a previously published report (Magiorakos et al., 2012). The MIC of CBF-2 were 950, 1050 and 1200 µg/ml for *P. aeruginosa* PAO1, *P. aeruginosa* RRLP1 and *P. aeruginosa* RRLP2, respectively. All further anti-QS analysis of CBF-2 was done at sub-MIC levels (0 - 900 µg/ml). The growth pattern of sub-MIC levels of CBF-2-treated organisms was studied through a dose-response growth curve study **(**Supplementary Figure 1). In spite of an extended lag phase, all the tested organisms exhibited similar growth pattern to that of the respective controls.

**CBF-2 at sub-MIC levels exhibited a significant anti-virulence property**

**Figure 3:**
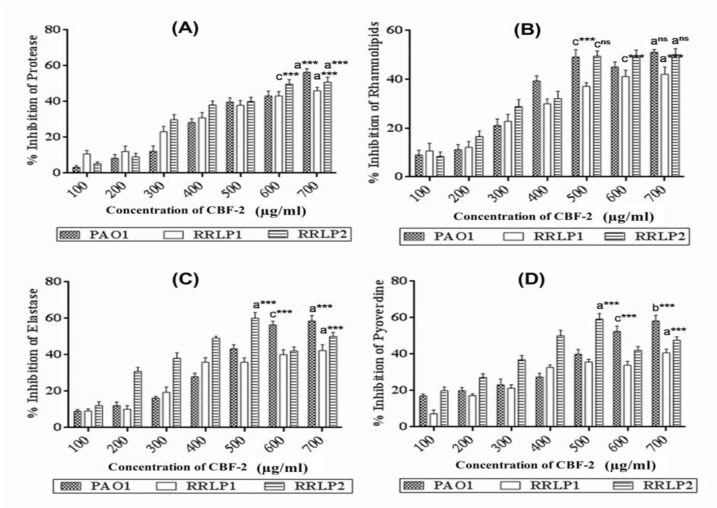
Influence of CBF-2 on quorum-regulated virulence factors and expression analysis of respective virulence genes and QS genes of tested organisms. (A) LasA protease (B) Rhamnolipid (C) LasB elastase and (D) Pyoverdine. The virulence factors estimation assays results were analyzed by two way ANOVA with Tukey’s multiple comparison posttest. Comparisons were performed in terms of the virulence factor production at different CBF-2 concentrations as ‘a’-500 µg/ ml vs 700 µg/ml, ‘b’-600 µg/ml vs 700 µg/ml, and ‘c’-500 µg/ml vs 600 µg/ml (***p<0.001, ns: not significant).

Quorum regulated virulence factors (VFs) like LasA protease, rhamnolipids, LasB elastase and pyoverdine play a major role in pathogenesis of uropathogenic *P. aeruginosa *(Sarabhai et al., 2013). In the present study, CBF-2 treatment notably depleted the foresaid VFs production in tested strains (Figure 3). Quantification of LasA protease (Figure 3A), revealed that CBF-2 at 700 µg/ml exhibited maximum protease depletion in PAO1 (56.34±2.1%) whereas, in clinical isolates (RRLP2 and RRLP1), the rate of inhibition was 49.82±2.8 and 46.13±2.45%, respectively (p<0.001). Among various concentrations tested, CBF-2 at 700 μg/ml, effectively inhibited the bio-surfactants production in the tested organisms (p<0.001; Figure 3B). Maximum reduction was observed in PAO1 (51.12±1.09 %), followed by RRLP2 (50.12±2.45%) and RRLP1 (42.12±2.98%). Treatment with CBF-2 exhibited maximum elastase inhibition in PAO1 (58.61±3.02%) and RRLP1 (40.38±3.0%) at 700 µg/ml (p<0.001) whereas, for RRLP2 the reduction was 60.19±2.83% (p<0.001) at 500 µg/ml (Figure 3C). Reduction in pyoverdine production by PAO1 was 58.21±3.06% with 700 µg/ml of CBF-2 (p<0.001). Among the clinical isolates, CBF-2 exhibited a significant drop in pyoverdine production by 40.74±2.01% (700 μg/ml) and 59.32±2.9% (500 μg/ml) in RRLP1 and RRLP2, respectively (p<0.001; Figure 3D).

**CBF-2 treatment reduced mRNA expression levels of virulence genes in tested organisms**

**Figure 4:**
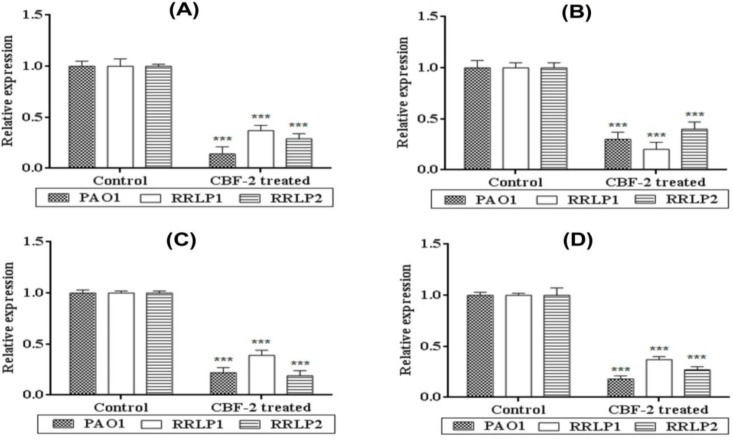
Influence of CBF-2 on the expression of virulence genes in tested organisms. (A) *lasA* (B) *rhlB *(C) *lasB* and (D) *pvdA*. Gene expression results were analyzed by two way ANOVA with Sidak’s multiple comparison posttest. Comparisons were performed between genes of CBF-2 untreated (Control) vs those of the CBF-2-treated ones (***p<0.001).

*In vitro* quantification results of virulence phenotypes correlated well with the cognate virulence gene expression pattern in all tested organisms (Figure 4). CBF-2 at 700 μg/ml significantly (p<0.001) reduced the relative gene expression of quorum-regulated VFs; *lasA* (encoding protease, Figure 4A), *rhlB* (encoding rhamnosyl transferase subunit B, Figure 4B), *lasB* (encoding elastase, Figure 4C), and *pvdA* (coding for pyoverdine, Figure 4D) in all tested organisms. Among the clinical isolates, RRLP1 exhibited reduced expression levels of 63, 80, 61 and 63% in comparison with RRLP2 which exerted expression level reductions of 71, 60, 81 and 73% for VFs genes (*lasA*, *rhlB*, *lasB *and *pvdA*, respectively); which significantly contribute to the virulence and pathogenicity. In PAO1, the reduction in the expression of genes was in the order of *lasA* (86%)>*pvdA *(82%)>*lasB* (70%)>*rhlB *(70%).

**CBF-2 treatment hampered the quorum sensing mechanism**

**Figure 5:**
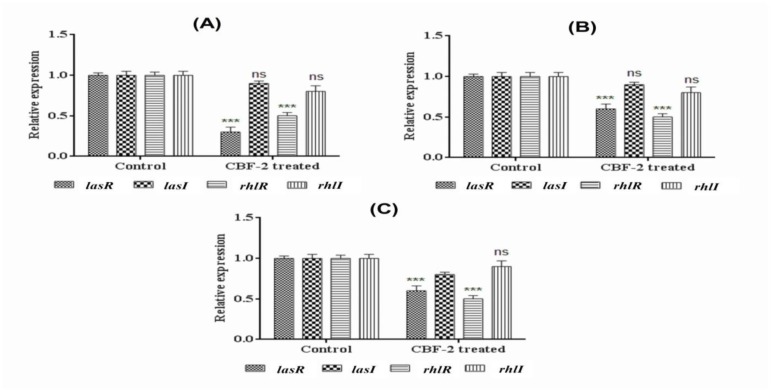
Influence of CBF-2 treatment on expression of quorum sensing genes (*lasR/I* and *rhlR/I*). (A) PAO1 (B) RRLP1 and (C) RRLP2. Gene expression results were analyzed by two way ANOVA with Sidak’s multiple comparison posttest. Comparisons were performed between genes of CBF-2 untreated (Control) vs those of the CBF-2-treated ones (***p<0.001).

Among the QS genes, test fraction (700 µg/ml) inhibited the expression of quorum receptor genes (*lasR* and *rhlR*; p<0.001) rather than the signal synthase genes (*lasI* and *rhlI*) (Figure 5). The inhibition was 77% and 45% (PAO1, Figure.5A), 68% and 62% (RRLP1, Figure 5B), and 88 and 77% (RRLP2, Figure. 5C) for *lasR* and *rhlR*, respectively. Similarly, reduction in the expression of *lasI *and *rhlI* was found to be 10 and 20% (PAO1), 10 and 20% (RRLP1), 20 and 10% (RRLP2), respectively.

**CBF-2 exhibited notable anti-biofilm and biofilm eradication property without bactericidal activity**

**Figure 6:**
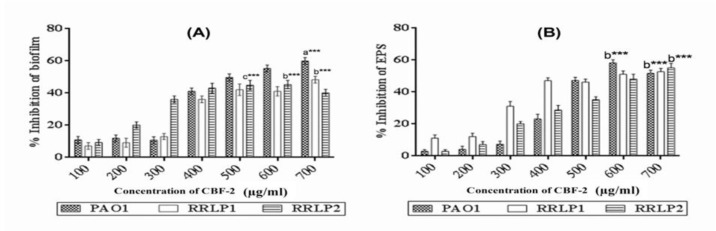
Effect of CBF-2 on biofilm formation, EPS production. (A) Biofilm and (B) EPS. The percentage inhibition of respective virulence factors are expressed as mean±SD (n=6). The results were analyzed by two way ANOVA with Tukey’s multiple comparison posttest. Comparisons were performed in terms of the virulence factor production at different CBF-2 concentrations as ‘a’- 500 µg/ ml vs 700 µg/ml, ‘b’- 600 µg/ml vs 700 µg/ml, and ‘c’- 500 µg/ml vs 600 µg/ml (***p<0.001).

Biofilm biomass and EPS production by the tested organisms after CBF-2 treatment were tested (Figure 6). Treatment with CBF-2, reduced the biofilm biomass of PAO1 and RRLP1 by 59.98±2.1 and 48.35±2.1%, respectively at 700 µg/ml (p<0.001) whereas, RRLP2 exhibited minimum biofilm production at 600 µg/ml (%inhibition: 45.32±1.9, p<0.001) (Figure 6A). EPS formed by the test organisms, with or without the active fraction was quantified. Obtained results (Figure 6B) presented a significant inhibitory action for CBF-2 upon EPS production in all tested organisms. Reduction in EPS production in PAO1 by CBF-2 (600 µg/ml) was found to be 58.21±2.7% (p<0.001). Among the clinical isolates, CBF-2 at 700 µg/ml (p<0.001) exhibited maximum EPS reduction by 52.73±2.19% (RRLP1) and 55.12±2.8% (RRLP2). 

**Figure 7:**
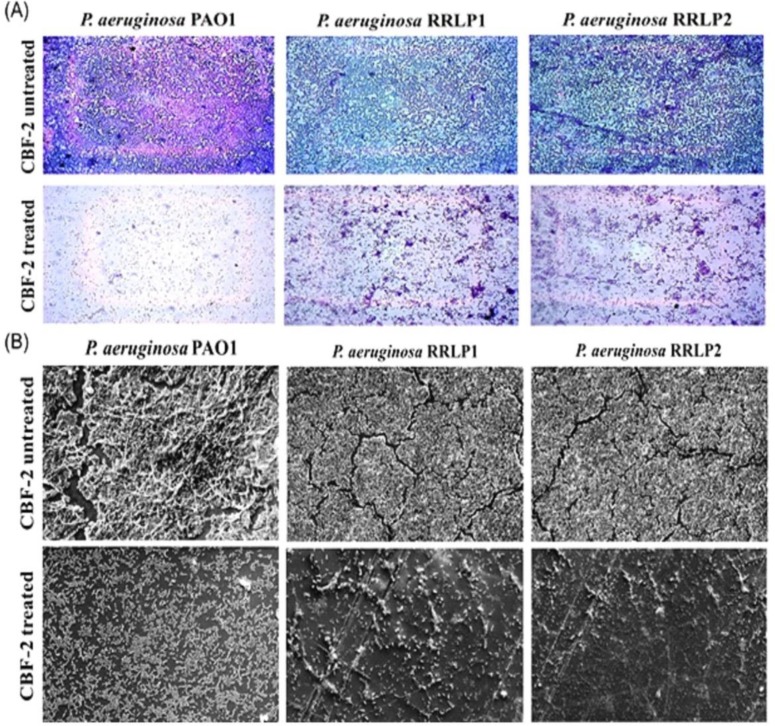
Microscopic images of CBF-2-treated and untreated biofilms. (A) Crystal violet staining (40X magnification), and (B) Scanning electron micrographs (bar: 20μm at 1500X magnification).

Furthermore, the microscopic evaluation of crystal violet-stained CBF-2-treated biofilms (PAO1 and RRLP1-700 µg/ml and RRLP2 - 600 µg/ml) was performed (Figure 7A). Microscopic analysis of stained biofilms revealed a well-developed thick layer of biofilm in the growth control (CBF-2 untreated) glass slides, which was stained easily with CV stain. However, CBF-2-treated slides exhibited a significant reduction in biofilm formation by the test organisms with a visible reduction in the number of microcolonies. Unlike the fully matured growth control biofilms, CBF-2-treated biofilms evidently exhibited significantly hampered macrocolony arrangement, loosened matrix formation and lesser microcolonies.

The ability of CBF-2 to prevent CAUTIs was evaluated by the biofilm eradication assay (Figure 7B). Preformed biofilms by the tested organisms on the silicon Foley catheter were treated with CBF-2 and processed for SEM analysis. SEM analysis clearly indicated that sub-lethal concentration of CBF-2 notably modulated biofilm formation. Control (without CBF-2 treatment) biofilms exhibited microbes embedded in complex extracellular matrix. *P. aeruginosa* PAO1 formed unique thread-like structure along with mushroom-like accumulation of EPS. However, CBF-2-treated PAO1 biofilms exhibited lesser cell density and lacked thread-like structures with reduced (or) no EPS encapsulated compartments. Unlike PAO1, though, clinical isolates’ biofilms were devoid of thread-like structures, catheter surfaces were densely packed with microbial cells in well-developed biofilms with water channels to distribute nutrients and other molecules. Interestingly, CBF-2-treated biofilms produced by clinical isolates exhibited less number of microbial cells attached together with significantly reduced EPS production.

Further, AO/EB dual staining of the CBF-2-treated biofilms cells was performed to differentiate live and dead cells, in which live cells stained green by AO and dead cells stained red by EB. The fluorescence micrographs clearly indicated green colored cells in all CBF-2-treated biofilms similar to the untreated biofilms (Packiavathy et al., 2012) (Supplementary Figure 2).

**CBF-2 is safe at all tested doses**

**Figure 8:**
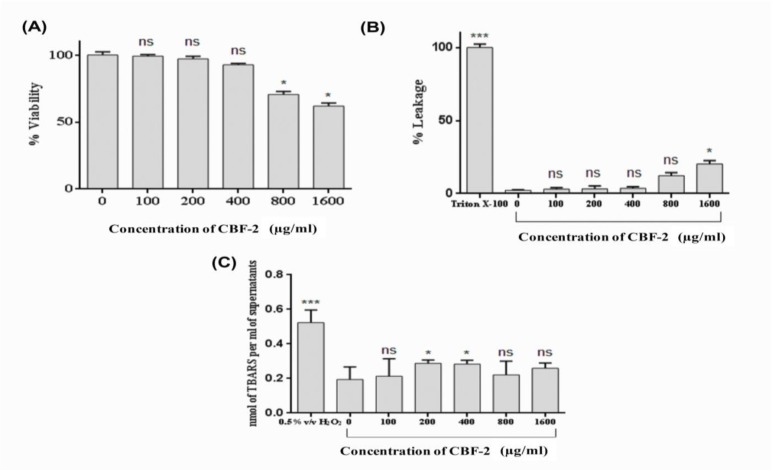
Cytotoxicity analysis of CBF-2. (A) MTT assay (B) LDH release and (C) Estimation of TBARS levels. The data was expressed as mean±SD (n=6). The results were analyzed by one way ANOVA with Dunnett’s multiple comparison posttest. Comparisons were performed vs Control (CBF-2, 0.0 μg/ml). (ns: not significant) (*** p<0.001 and *p<0.05).

In the present study, *in vitro* toxicity profiling of CBF-2 was performed (Figure 8). Though more than 50% of the cells were found to be viable even after treatment with the highest level of CBF-2 (1600 μg/ml), a dose-dependent reduction in cell viability was observed following treatment with the test compound (Figure 8A). The cell viability associated with the lowest and the highest test compound concentration was found to be 99.27±1.01% (100 μg/ml) and 61.9±2.3% (1600 μg/ml), respectively. Effect of CBF-2 treatment on the leakage of lactate dehydrogenase (LDH) was evaluated, which exhibited a dose-dependent % leakage of LDH. However, the release was incomparable with that of the positive control (Triton X-100) which exhibited maximum levels of LDH (p<0.001). Among the CBF-2 doses, maximum LDH leakage was found at the dose of 1600 𝜇g/ml (20.34±2.3%), while the dose of 100 𝜇g/ml exhibited a minimum release of 2.27±0.32%, in comparison with positive control (Figure 8B). Unlike the previous cytotoxicity assays, quantification of TBARS did not indicate a dose-dependent pattern. In comparison with the positive control (i.e. H_2_O_2_), which exhibited maximum TBARS level (0.523±0.083 nmol per ml of supernatant, p<0.001), none of the tested doses produced a significant elevation in the levels of TBARS (an average of 0.253±0.051 nmol per ml of supernatant) in comparison with the control (0.194±0.073 nmol per ml of supernatant) as presented in Figure 8C.

## Discussion

*Syzygium aromaticum* (clove), being one of the highly beneficial spices to humans health, studies have evaluated the anti-QS property of clove bud extract(s) (Krishnan et al., 2012 ▶) and clove oil (Khan et al., 2009 ▶) by estimating the reduction in quorum-controlled violacein pigment in biomarker strains. But these studies failed to validate its anti-QS characteristics against clinical isolates to evaluate the clinical efficacy and efficiency. In this regard, we studied the anti-virulence potency of partially purified *S. aromaticum* bud extract(s)/fraction(s) against two clinical isolates of *P. aeruginosa* (i.e. RRLP1 and RRLP2) from patients with catheter-associated urinary tract infection using bioactivity-guided partial purification scheme (Figure 1). Organic extracts of plants *viz* methanol extract of *Terminalia chebula* fruits, methanolic extract of *Hyptis suaveolens*, and ethanol extracts of *Mangifera indica* and *Punica granatum, *have shown notable anti-QS activity (Sarabhai et al., 2013 ▶, Salini et al., 2015 ▶, Viswanathan et al., 2015 ▶). Similarly, in the present study, during the in initial readout assays, methanol extract(s)/fraction(s) exhibited greater anti-QS activity (Table 1), suggesting that the effective phytochemical compound(s) might have partitioned more in organic phase, as observed in the previous observations (Sarabhai et al., 2013 ▶). GC-MS analysis of the most active fraction (i.e. CBF-2), isolated after size exclusion chromatography, confirmed the presence of eugenol, as the major bioactive compound (Figure 2). *S. aromaticum* showed the highest content of polyphenols and presence of these phytochemicals, especially eugenol as an organic phenol, attributed to a wide range of medicinal properties (Kong et al., 2014 ▶).

Quorum-regulated VFs like toxins, siderophores, biosurfactants, proteases, biofilms and EPS are regarded as indicators of the optimal QS regulation and aid in virulent uropathogenic properties of *P. aeruginosa *(Mittal et al., 2009, Sarabhai et al., 2013 ▶). LasA staphylolytic protease (20 kDa) is a zinc metalloendopeptidase produced by *P. aeruginosa *which is believed to play a major role in host tissue degradation (Packiavathy et al., 2012 ▶). Being a biosurfactant, rhamnolipid protects bacterial cells against phagocytosis and helps to thrive in host system (Packiavathy et al., 2012 ▶). Bacterial elastase has been shown to cause proteolytic damages to the tissues and other major components involved in the phagocytosis. Siderophores like pyoverdine chelates the bound iron from host for optimal growth and helps in pathogen survival in host milieu (Mittal et al., 2009 ▶, Adonizio et al., 2008 ▶). Reduction in these VFs’ production indicates the anti-virulent property of the tested compound through QS inhibition mechanism. Various studies have tested these VFs to evaluate the anti-virulence property of the test extract(s)/compound(s) (Mittal et al., 2009 ▶, Sarabhai et al., 2013 ▶, Adonizio et al., 2008 ▶, Packiavathy et al., 2012 ▶). Corroborating well with these results, in the present study, treatment with sub-MIC levels of CBF-2 depleted the VFs in all the tested strains at various concentrations, significantly at 700 µg/ml (Figure 3). The literature indicate that QS inhibitors obtained from several common food products down-regulated major quorum-regulated VFs like *rhlAB*, *lasA*, *chiC*, *lasB, *etc. in PAO1 (Jakobsen et al., 2012 ▶). *In vitro* attenuation of virulence phenotypes correlated well with the cognate virulence gene (*lasA*, *rhlB*, *lasB* and *pvdA*) expression, confirming the transcriptional level inhibition after CBF-2 (700 µg/ml) treatment (Figure 4).

Reduction in biofilm production has been the gold standard for screening anti-QS compounds (Annapoorani et al., 2013 ▶, Husain et al., 2013 ▶). Evaluation of QS inhibitory property of clove oil and curcumin have exhibited significant reduction in biofilm production by reporter strains and indicated a direct correlation between EPS production and biofilm complexity (Husain et al., 2013, Yates et al., 2002). Parallel to these reports, in the present study, CBF-2 treatment indicated a similar link between EPS (Figure 6A) and biofilm biomass (Figure 6B) production. Hence, CBF-2 may diminish biofilm formation by the test organisms through interfering with their ability to produce EPS. Considering the role of biofilms and related pathogens in catheter-associated urinary tract infections, compound(s) with biofilm eradication property has been considered vital (Annapoorani et al., 2013 ▶, Husain et al., 2013 ▶). SEM analysis of CBF-2-treated catheter biofilms demonstrated morphologically simplified biofilms, which ultimately led to rapid drug penetration and/or microbial clearance by the host immune system (Mittal et al., 2009).

To understand the possible QSI mechanism of CBF-2, gene expression analysis of QS genes (namely, *lasR*/*I* and *rhlR*/*I*) was performed in all tested pathogens (Figure 5). Generally, QS inhibition can be achieved via autoinducer analogue-receptor interactions or by enzymatic degradation of signal molecules (Ni et al., 2009 ▶). In fact, degradation of signal molecules is pH-dependent and as the pH of the test fraction was neutral, there were no possibilities for the spontaneous inactivation of autoinducers (Yates et al., 2002 ▶). Results obtained in the present study suggested that the QS inhibitory action of CBF-2 probably resulted from the reduction in the capacity of LasR type transcription factors to perceive their cognate signal molecules linked with a consequent reduction of the quorum-controlled VFs genes and the signal synthase genes (Kalia, 2013 ▶, Kumar et al., 2015 ▶) (corroborated by the down-regulation of the expression of *lasI* and *rhlI* genes in comparison with the *lasR* and *rhlR* genes). Furthermore, a detailed mechanistic study should be performed with phytocompounds in CBF-2 to understand the exact mechanisms underlying its anti-virulence property. 

Scientific investigations have already proved that QS inhibition has no effect upon cell growth and viability, thus minimizing the risk of resistance development (Sarabhai et al., 2013 ▶, Adonizio et al., 2008 ▶, Annapoorani et al., 2013 ▶, Ni et al., 2009 ▶). In the present study, all the anti-virulence and anti-biofilm assays were carried out at sub-MIC levels of CBF-2. Dose-response growth study of CBF-2-treated test organisms was done (Supplementary Figure 1) and in spite of an extended lag phase, all tested organisms exhibited unaffected growth pattern, compared to the respective growth controls. Similarly, AO/EB dual staining confirmed the viability of the CBF-2-treated biofilm cells in comparison to the control biofilm cells (Supplementary Figure 2). These results clarify the effect of CBF-2 doses on cellular growth and proliferation and conclude quorum-sensing inhibition as the plausible mechanism underlying the observed anti-virulence property.

Phyto-compound(s) present in CBF-2 can modify and/or interfere with basic cellular functions and cell viability (Kampa et al., 2007 ▶). Hence, in the present study, *in vitro* toxicity of CBF-2 was investigated (Figure 8). The observed dose-dependent reduction in cell viability induced by the test compound in the MTT assay (Figure 8A) might be due to the presence of phenol moiety and hydrophobic nature of polyphenol constituents in CBF-2 that might be the reason for the observed dose-dependent reduction in cell viability of macrophages (Unnikrishnan et al., 2014 ▶, Galati and O'Brien,2004 ▶). Results of the quantification of lactate dehydrogenase (LDH), as an indicator of irreversible cell death due to cell membrane damage, showed similar pattern with that of the MTT assay (Figure 8B). Previously, the cytotoxicity analysis of 4 *Staphylea* spp. (*S. colchica*, *S. elegans*, *S. holocarpa* and *S. pinnata*) upon A431 carcinoma cells exhibited a dose- and time-dependent increase in LDH release, substantiating the observations of the present study (Janero, 1990). Increased hydrophobicity of CBF-2 at higher concentrations causes cell blebbing and shrinkage, by lipid bi-layer solubilization (Galati and O'Brien, 2004 ▶) followed by elevated membrane permeability which might result in LDH leakage into the medium, as observed in the present study.

Oxidation of membrane lipids is considered as one of the main mechanisms involved in the deterioration of cellular architecture that eventually leads to cell death, when cells are exposed to various stress conditions (abiotic or biotic) (Bhattacharjee, 2014 ▶). Hence, quantification of TBARS levels (Figure 8C), an indicator of lipids oxidation, was performed to assess the possibility of reactive oxygen species (ROS)-mediated lipid peroxidation and associated membrane damage (Janero, 1990 ▶). Polyphenols, the major phytochemical constituent(s) of CBF-2, have been widely accepted as anti-oxidant agents and also were shown to have protective effects against lipid peroxidation, through radical scavenging activities (Kong et al., 2014, Cortés-Rojas et al., 2014). Hence, authors believe that, these components might have prevented ROS-mediated lipid peroxidation, deprecating ROS independent mechanism of membrane damage, which could be the only mechanism underlying the persistent level of TBARS after CBF-2 treatment. However, the possibility of quiononemethide (QM) generation and QM-mediated toxicity alongside the elevated hydrophobicity could not be neglected as a reason for the observed reduction in cell viability (Cortés-Rojas et al., 2014 ▶, Thompson et al., 1998 ▶).

There are evidences detailing the ability of clinical strains to evolve resistance towards anti-virulence agents; those proved to be effective against engineered reporter strains and laboratory reference strains. Therefore, it is necessary to testthese canonical agents against clinical pathogens. Hence, the present study evaluated the anti-virulence potency of partially purified *S. aromaticum* bud fraction(s), against clinical isolates from patients with catheter-associated urinary tract infection, for the first time. The results of the present study indicated that eugenol-rich clove bioactive fraction-2 (CBF-2) has potent anti-virulence and anti-biofilm activity against biofilm-associated clinical isolates besides the model organism, *P. aeruginosa* PAO1. Future studies are needed to examine its active component(s) to be introduced as a cleaning agent for hospital gadgets, surfaces and/or coating catheter surfaces to prevent nosocomial infections.

## References

[B1] Abraham SVPI, Palani A, Ramaswamy BR, Shunmugiah KP, Arumugam VR (2011). Antiquorum sensing and antibiofilm potential of Capparis spinosa. Arch Med Res.

[B2] Adonizio A, Kong KF, Mathee K (2008). Inhibition of quorum sensing-controlled virulence factor production in Pseudomonas aeruginosa by South Florida plant extracts. Antimicrob Agents Chemother.

[B3] Annapoorani A, Kalpana B, Musthafa KS, Pandian SK, Ravi AV (2013). Antipathogenic potential of Rhizophora spp against the quorum sensing mediated virulence factors production in drug resistant Pseudomonas aeruginosa. Phytomedicine.

[B4] Bhattacharjee S (2014). Membrane lipid peroxidation and its conflict of interest: the two faces of oxidative stress. Curr Sci.

[B5] Cortés-Rojas DF, de Souza CRF, Oliveira WP (2014). Clove (Syzygium aromaticum): a precious spice. Asian Pac J Trop Biomed.

[B6] Ćurčić MG, Stanković MS, Mrkalić EM, Matović ZD, Banković DD, Cvetković DM, Đačić DS, Marković SD (2012). Antiproliferative and proapoptotic activities of methanolic extracts from Ligustrum vulgare L as an individual treatment and in combination with palladium complex. Int J Mol Sci.

[B7] Galati G, O'Brien PJ (2004). Potential toxicity of flavonoids and other dietary phenolics: significance for their chemopreventive and anticancer properties. Free Radic Biol Med.

[B8] García-Contreras R, Maeda T, Wood TK (2013). Resistance to quorum-quenching compounds. ApplEnviron Microbiol.

[B9] García-Contreras R, Peréz-Eretza B, Jasso-Chávez R, Lira-Silva E, Roldán-Sánchez JA, González-Valdez A, Soberón-Chávez G, Coria-Jiménez R, Martínez-Vázquez M, Alcaraz LD (2015). High variability in quorum quenching and growth inhibition by furanone C-30 in Pseudomonas aeruginosa clinical isolates from cystic fibrosis patients. Pathog Dis.

[B10] Gibot S (2004). Fighting the enemy properly?. Crit Care Med.

[B11] Husain FM, Ahmad I, Asif M, Tahseen Q (2013). Influence of clove oil on certain quorum-sensing-regulated functions and biofilm of Pseudomonas aeruginosa and Aeromonas hydrophila. J Biosci.

[B12] Jakobsen TH, Bragason SK, Phipps RK, Christensen LD, van Gennip M, Alhede M, Skindersoe M, Larsen TO, Høiby N, Bjarnsholt T (2012). Food as a source for QS inhibitors: iberin from horseradish revealed as a quorum sensing inhibitor of Pseudomonas aeruginosa. Appl Environ Microbiol.

[B13] Janero DR (1990). Malondialdehyde and thiobarbituric acid-reactivity as diagnostic indices of lipid peroxidation and peroxidative tissue injury. Free Radic Biol Med.

[B14] Kalia VC (2013). Quorum sensing inhibitors: an overview. Biotechnol Adv.

[B15] Kampa M, Nifli AP, Notas G, Castanas E (2007). Polyphenols and cancer cell growth. Reviews of physiology, biochemistry and pharmacology.

[B16] Khan MSA, Zahin M, Hasan S, Husain FM, Ahmad I (2009). Inhibition of quorum sensing regulated bacterial functions by plant essential oils with special reference to clove oil. Lett Appl Microbiol.

[B17] Kong X, Liu X, Li J, Yang Y (2014). Advances in pharmacological research of eugenol. Curr Opin Complement Alternat Med.

[B18] Krishnan T, Yin WF, Chan KG (2012). Inhibition of quorum sensing-controlled virulence factor production in Pseudomonas aeruginosa PAO1 by Ayurveda spice clove (Syzygium aromaticum) bud extract. Sensors.

[B19] Kumar L, Chhibber S, Kumar R, Kumar M, Harjai K (2015). Zingerone silences quorum sensing and attenuates virulence of Pseudomonas aeruginosa. Fitoterapia.

[B20] Magiorakos AP, Srinivasan A, Carey R, Carmeli Y, Falagas M, Giske C, Harbarth S, Hindler J, Kahlmeter G, Olsson‐Liljequist B (2012). Multidrug‐resistant, extensively drug‐resistant and pandrug‐resistant bacteria: an international expert proposal for interim standard definitions for acquired resistance. Clin. Microbiol Infect.

[B21] Mittal R, Aggarwal S, Sharma S, Chhibber S, Harjai K (2009). Urinary tract infections caused by Pseudomonas aeruginosa: a minireview. J Infect Public Health.

[B22] Ni N, Li M, Wang J, Wang B (2009). Inhibitors and antagonists of bacterial quorum sensing. Med Res Rev.

[B23] Packiavathy IASV, Agilandeswari P, Musthafa KS, Pandian SK, Ravi AV (2012). Antibiofilm and quorum sensing inhibitory potential of Cuminum cyminum and its secondary metabolite methyl eugenol against Gram negative bacterial pathogens. Food Res Int.

[B24] Rathinam P, Viswanathan P (2014). Effects of antibiotics upon quorum sensing regulated characters: a propitious scheme against device associated infections. Int J Pharm Pharm Sci.

[B25] Salini R, Sindhulakshmi M, Poongothai T, Pandian SK (2015). Inhibition of quorum sensing mediated biofilm development and virulence in uropathogens by Hyptis suaveolens. A Van Leeuw J Microb.

[B26] Sarabhai S, Sharma P, Capalash N (2013). Ellagic acid derivatives from Terminalia chebula Retz Downregulate the expression of quorum sensing genes to attenuate Pseudomonas aeruginosa PAO1 virulence. PLoS One.

[B27] Thompson DC, Barhoumi R, Burghardt RC (1998). Comparative toxicity of eugenol and its quinone methide metabolite in cultured liver cells using kinetic fluorescence bioassays. Toxicol Appl Pharmaco.

[B28] Unnikrishnan P, Suthindhiran K, Jayasri M (2014). Inhibitory potential of Turbinaria ornata against key metabolic enzymes linked to diabetes.

[B29] Van Delden C, Iglewski BH (1998). Cell-to-cell signaling and Pseudomonas aeruginosa infections. Emerg Infect Diseases.

[B30] Viswanathan P, Rathinam P, Suneeva S (2015). Plant Quorum Sensing Inhibitors: Food, Medicinal Plants, and Others. Quorum Sensing vs Quorum Quenching: A Battle with No End in Sight.

[B31] Wikler M (2009). Clinical and Laboratory Standards Institute. Methods for dilution antimicrobial susceptibility tests for bacteria that grow aerobically; approved standard.

[B32] Yates EA, Philipp B, Buckley C, Atkinson S, Chhabra SR, Sockett RE, Goldner M, Dessaux Y, Cámara M, Smith H (2002). N-acylhomoserine lactones undergo lactonolysis in a pH-, temperature-, and acyl chain length-dependent manner during growth of Yersinia pseudotuberculosis and Pseudomonas aeruginosa. Infect immun.

